# Asynchronous Assessment of the Relative Intensity of an Online Dance for Parkinson’s Class Using Heart Rate and Video Data

**DOI:** 10.3390/s26217075

**Published:** 2026-11-09

**Authors:** Aston K. McCullough, Nandini Erodula, Arthur F. Kramer, Linda Tickle-Degnen, David Leventhal

**Affiliations:** 1 Laboratory for the Scientific Study of Dance, Institute for Cognitive and Brain Health, Northeastern University, Boston, MA 02115, USA; n.erodula@northeastern.edu; 2 Department of Physical Therapy, Movement & Rehabilitation Sciences, Bouvé College of Health Sciences, Northeastern University, Boston, MA 02115, USA; 3 Department of Music, College of Art, Media & Design, Northeastern University, Boston, MA 02115, USA; 4 Beckman Institute, University of Illinois Urbana-Champaign, Champaign, IL 61820, USA; afkramer@illinois.edu; 5 Department of Occupational Therapy, Tufts University, Medford, MA 02155, USA; linda.tickle_degnen@tufts.edu; 6 Dance for PD, Mark Morris Dance Group, Brooklyn, NY 11217, USA; david@danceforpd.org

**Keywords:** physical activity, machine learning, detection algorithm

## Abstract

Background: Regular participation in physical activity (PA) is positively associated with greater functional independence among adults living with Parkinson’s Disease (PD); however, little is known about the dose of PA that adults receive during dance classes specifically designed for adults with PD. Therefore, the purpose of this study was to evaluate the relative PA intensity of Dance for PD^®^ classes that are conducted online and to preliminarily calibrate a multimodal algorithm for detecting the PA intensity of online, group-based Dance for PD^®^ classes using heart rate and 2D video data. Methods: Adults with a diagnosis of idiopathic PD were recruited as participants; a total of n=15 adults with PD [ages 46 to 84 years; female 93.3% (14)] completed this study. Each participant wore a wireless heart rate (HR) monitor during two ≈60 min group-based, online Dance for PD^®^ classes that were held on separate days, and HR data were recorded asynchronously. A signal processing and unsupervised machine learning pipeline was implemented to align HR data across participants and automatically differentiate group-level PA intensity using HR data. Results: HR responses were found to be stable (ICC: 0.93) across exposures to the same online dance class. The average relative intensity of Dance for PD^®^ classes was 65% HR_max_ (range 48% to 78%) during the initial Dance for PD^®^ class, and the mean within-subjects difference in the relative PA intensity between classes was 0.67% (2.9) HR_max_. The novel pipeline for classifying lower- versus higher-intensity PA segments resulted in a segmentation solution that successfully distinguished respective lesson activities quantitatively and qualitatively using heart rate and video data. Conclusions: An online, group-based Dance for PD^®^ class elicited a moderate PA intensity among middle-aged to older adults living with PD at the group level. A novel multimodal signal analysis pipeline successfully distinguished between lower and higher PA intensity behaviors at the group level using HR data, in addition to providing rich qualitative descriptions about the motor behaviors respectively associated with each PA intensity class using video data.

## 1. Introduction

Parkinson’s disease (PD) is a neurodegenerative disorder that affects an estimated 1 million people in the United States [1]. The onset of PD symptoms typically occurs after the age of 50, and data show that the prevalence of PD increases with age, as approximately 0.3% of 50-year-olds, 1–2% of 65-year-olds, and 4–5% of 85-year-olds in the U.S. are reported to be living with PD [2]. PD is more prevalent in men [3], and adults living with PD experience motor impairments such as tremor, bradykinesia (slowness of movement), dystonia (painful muscle cramps), stiffness, and freezing of gait [1,4,5]. Approximately 40% of individuals with PD also experience at least mild cognitive impairment [6]. Studies have shown that engaging in physical activity (PA) may delay the progression of PD symptoms [7], and a recent meta-analysis revealed that engaging in dance, when compared to other modes of PA behavior, confers especial protective benefits across motor outcomes, such as balance, functional mobility, and cognitive function [8]. Though most of these studies have reported dance class duration and the frequency at which participants attended dance classes, the intensity of the PA in which participants engaged has been largely unreported in analyses of dance and health among adults living with PD. Thus, little is known about the PA intensities of the dance behaviors that led to most of these reported outcomes in the literature.

Studies on long-term PA exposures provide evidence that the dose of a PA exposure is a key factor in estimating its impact on health [9]. PA dose (e.g., total energy expenditure) may be defined in terms of PA intensity, frequency, and duration—each of which are known to differentially contribute to the impact PA confers on physiological, psychological, and cognitive health outcomes [10,11,12,13,14,15]. Most prior studies that have used wearable sensors to measure dance training exposures have been limited to adopting measurement design strategies that, while appropriate for quantifying the dose of PA behaviors such as walking, may not be sufficient for measuring dance behavior. For example, studies using accelerometry to quantify PA intensities during dance exposures have largely used hip-worn device placements [16]. However, dance is widely known to include repeated sets of stationary higher-intensity PA, such as battement (i.e., large, sweeping kicks), that are likely underestimated by a hip-worn device [17]. Moreover, resting energy expenditure has been shown to increase with disease progression among adults with PD [18], and adults with PD may exhibit increased energy expenditure during PA [19], which further points to the need for more refined methods for quantifying PA intensity during dance within this population group. A recent study on dance conducted among apparently healthy older adults showed that the use of data from a heart rate sensor greatly improved energy expenditure prediction accuracy during structured dance [20], and a study on free-form dance among 18- to 83-year-old adults reported that adults engaged in an average 74.3% of their heart rate reserve (i.e., a vigorous PA intensity) during 5 min dance bouts [21]. Moreover, studies have shown that the intensity of dance may vary by dance style [10] or performance context [22]. Given that PA dose is associated with myriad health outcomes [11], along with the wealth of prior work that shows participating in dance improves health and well-being among adults with PD, the dearth of available methods for measuring the PA intensity of dance among adults with PD underscores the need for sensor-based methods that are especially calibrated for measuring PA intensity within this population group.

Dance for PD^^®^^, a codified dance program for adults living with PD, is a widely accepted and safe PA modality for older adults with PD [23,24]. During Dance for PD^®^ sessions, participants are guided by a certified instructor to dance with musical accompaniment while seated in a chair, standing, and ambulatory [23]. Current PA recommendations for adults with PD suggest engaging in PA at an intensity of ≥60% $\dot{V}$O_2 max_ (i.e., at least a moderate PA intensity) for 150 min per week in order to receive the health-enhancing benefits of PA [25]. Accelerometry has been widely used to quantify PA intensity among individuals with PD [26,27], and the online delivery of Dance for PD^®^ classes is known to be feasible and acceptable. However, it is unknown to what extent participation in online Dance for PD^®^ lesson activities elicits consistent relative PA intensities among participants, and no prior study has directly monitored the relative PA intensity of online Dance for PD^®^ classes using heart rate sensors. Dance for PD^®^ classes offer participants an adaptive dance curriculum within which individuals are invited to engage in bouts of free-form dance, which are performed alongside otherwise structured action sequences. Though Dance for PD^®^ classes typically progress from seated activities to ambulatory activities within a single session, participants are invited to remain seated while dancing at any time during a given session, which means that the relative PA intensity may differ widely across participants within a Dance for PD^®^ class. Dance for PD^®^ Online blends structured dance and free-form dance throughout most sessions, and no prior studies have confirmed the PA intensity of these accessible online classes. To advance measurement methods for quantifying the PA intensity of online Dance for PD^®^ classes, additional research that uses wearable sensors to quantify the relative PA intensity of Dance for PD^®^ classes is needed.

In contrast to in-person dance classes, online dance classes are accessible to a wider number of people who may otherwise experience barriers to travel that would limit attendance at in-person dance classes; however, additional methods are required to quantify the PA intensity of online dance classes. Despite strong evidence that supports the practice of dance for improved well-being among adults with PD, there remains a specific need for sensor-based methods that quantify the PA intensity of online Dance for PD^®^ classes, especially accounting for reports that suggest PD progression, age, and dance style may be important factors to consider when monitoring PA intensity during dance. Therefore, this study intended to (1) determine the stability of heart rate responses to an online Dance for PD^®^ class among a cohort of adults living with PD, and (2) automatically identify and characterize relatively lower- and higher-intensity lesson plan activities in a recorded online Dance for PD^®^ class using heart rate data that were collected asynchronously.

## 2. Materials and Methods

Site and Sample. Participants were recruited from across the United States via word of mouth, flyers, and emails that were distributed to dance centers, community centers, and among the network of Dance for PD^®^ certified teachers from July 2024 to May 2025. Participants confirmed they were without contraindication to exercise by completing the Physical Activity Readiness Questionnaire for Everyone [28]. All participants confirmed they were currently taking Dance for PD^®^ classes and that their physician had not informed them of having a score >3 on the Hoehn & Yahr scale prior to enrolling in this study. The protocol was approved by the Northeastern University IRB (Protocol #23-10-30), and all participants (N=19) provided verbal and written informed consent. Data were collected between July 2024 and June 2025.

Procedures. After giving their informed consent, each participant was asked to provide sociodemographic information along with information about the date they received a diagnosis of PD and any medications taken. Participants were then asked to inform the study team which size chest strap the lab should send them to use with a wireless heart rate monitor. The lab then sent each participant a kit with a wireless heart rate monitor (along with their selected chest strap size) and a tablet. A member of the lab team then scheduled an individualized orientation session with each participant to help them learn to operate the device; each orientation session lasted approximately 30 min in duration. During the orientation session, the lab member helped each participant initialize the tablet, put on the heart rate monitor, initialize a heart rate detection session to measure resting heart rate, appropriately close the heart rate measurement session on the tablet, and finally confirm that the session was saved locally. Participants were then invited to sign up to attend a total of two (2) ≈60 min dance classes on two separate days; the lesson content and teacher featured within the ≈60 min pre-recorded dance class was identical across both sessions. Participants were asked to put on the heart rate monitor prior to the start of each ≈60 min Dance for PD^®^ session, and each participant met with a member of the study team to confirm the devices were functioning properly. Each online Dance for PD^®^ session was monitored by a certified Dance for PD^®^ teacher along with a team member from the lab who engaged in the session as a participant observer. HR data were downloaded from the cloud after each session. After completing the dance session, participants returned the heart rate monitoring kit to the lab in the mail. Participants received a $60 gift card at the end of the study.

Attendance and Field Notes. The research team determined session attendance via REDCap [29] for each participant. Field notes were maintained throughout this feasibility study to document participant reports on the acceptability of delivering the physiological measurement kit to each participant’s home, ease of kit setup, and the ease of using the chest-worn heart rate monitors during Dance for PD^®^ classes.

Sociodemographic Information. A brief sociodemographic questionnaire was used to collect data on participant age, sex, and race/ethnicity.

Medication Use and Brief Health History. The year of PD diagnosis was self-reported, along with medication use, using an antiparkinsonian medication questionnaire [30].

Activities of Daily Living. The Community Healthy Activities Model Program for Seniors (CHAMPS) questionnaire was used to quantify the number of activities of daily living participants completed during a typical week via self-report [31].

**Figure 1:**
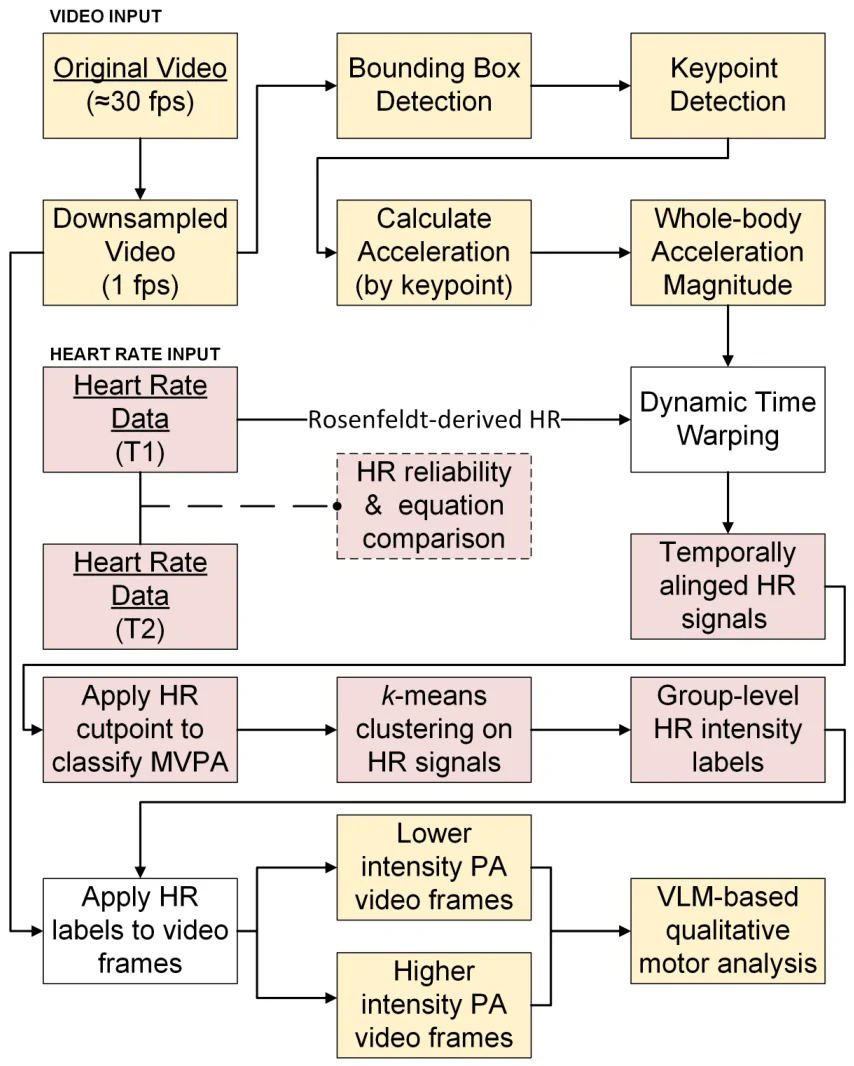
An overview of the multimodal data processing pipeline for using 2D video and heart rate data to detect group-level physical activity intensities and the associated motor behaviors during online lesson activities. The figure highlights inputs (underlined text) and outputs for video (light orange) and heart rate data (light red), along with multimodal signal integration points (white boxes) within the pipeline. Dotted lines correspond to heart rate reliability analyses. Abbreviations: frames per second (fps), measurement time points (T1 and T2), heart rate (HR), moderate-to-vigorous physical activity (MVPA), physical activity (PA), vision–language model (VLM).

Heart rate (HR). Polar wireless HR monitors (Polar, Bethpage, NY, USA) were used to collect HR signals during PA across both sessions, as indicated in Figure 1. Data were stored using the Polar Flow app, which was downloaded on the iPad for participants before deploying the kits. Before each Dance for PD^®^ session, a member of the lab met with every participant to ensure they were able to initialize a HR recording session on the iPad provided in the kit via the Polar App for iOS.

HR was converted into %HR_max_ using the equation by Rosenfeldt and colleagues [32], HR_max_: 166 − (1.15 × age) + (0.60 × HR_rest_). Heart rate was additionally converted into %HR_max_ using the equation by Gellish and colleagues [33], HR_max_: 207 − (0.70 × age). Across the heart rate results obtained using each respective equation, a cut point of ≥64%HR_max_ was applied to identify moderate-to-vigorous PA intensity epochs [34].

Kinematic analyses. The ≈60 min prerecorded Dance for PD^®^ class was analyzed using MATLAB R2025a in order to extract kinematic data from the teacher in the recording (Figure 1). The original video (854 × 480 pixels; 29.97 fps) was 59 min and 53 s in duration, and the entire video was downsampled to 1 fps by taking the first frame at each second. For each frame *k*, YOLOv4 [35] was applied in order to generate a bounding box around the person detected in each frame. Within the bounding box detected for any given frame, keypoints ($x_{k,j}$, $y_{k,j}$) were extracted for j∈{*right eye, left eye, nose, right ear, left ear, right shoulder, left shoulder, right wrist, left wrist, right elbow, left elbow, right hip, left hip, right knee, left knee, right ankle, left ankle*} locations using HRNet-W32 [36]. HRNet-W32 is available as a pretrained network for keypoint detection in MATLAB. If any keypoint was found to be within 20 pixels of the bounding box limits, YOLO4 was used to detect a new bounding box; otherwise, the bounding box from the prior frame k−1 was conserved. The final array of keypoints was then transformed into an array of whole-body acceleration vector magnitude values using the following steps:

1. For each respective vector of body segment keypoints ($x_{k,j}$, $y_{k,j}$), second-order finite differences were calculated
  (1) $$\left(x_{k,j}-2x_{k-1,j}+x_{k-2,j},\;y_{k,j}-2y_{k-1,j}+y_{k-2,j}\right)\;\mathrm{for}\;k\in \left[3,3593\right],$$
  which resulted in 3591 × 17 arrays of acceleration values $A_{xk,j}$ and $A_{yk,j}$ in units pixels/s^2^.
2. The arrays from step 1 $A_{xk,j}$ and $A_{yk,j}$ were zero padded to replace the first two epochs that were required to calculate the second-order finite differences, resulting in 3593 × 17 arrays of acceleration values.
3. The acceleration vector magnitude for each body segment *j* was then calculated over all frames as $A_{\mathrm{mag}k,j}=\sqrt{\left(A_{xk,j}⊙A_{xk,j}\right)+\left(A_{yk,j}⊙A_{yk,j}\right)}$. To replace the zero-padded values for the first two epochs from step 2, the nearest non-zero value (i.e., the acceleration vector magnitude at k=3) was imputed.
4. The geometric mean $m_{k}={\left[\prod_{j=1}^{17}A_{\mathrm{mag}k,j}\right]}^{\frac{1}{17}}$ was then calculated for each frame to obtain a 3593 × 1 vector of mean whole-body acceleration values, and a median filter with a 5 epoch window was applied to smooth $m_{k}$.

The final 3593 × 1 vector $\hat{c}$ of smoothed acceleration values represented the teacher’s whole-body kinematic data for the duration of the approximately 60 min video.

Multimodal Signal Processing. Because participants were instructed to begin recording HR data before the video started and stop the HR recording after the video ended in each session, the length of each HR recording differed across participants. As such, temporal alignment between the kinematic and HR data was required (Figure 1). Dynamic Time Warping (DTW) [37] was used to align the acceleration trace $\hat{c}$, as generated from the video of the Dance for PD^®^ teacher, with each participant’s heart rate signal $U_{v}$. To define the optimal path between $\hat{c}$ and $U_{v}$ for each participant *v*, the Euclidean distance

(2) $$d_{fg}\left(\hat{c},U_{v}\right)=\sqrt{\sum_{z=1}^{Z}\left(c_{z,f}-u_{z,g}\right)\times \left(c_{z,f}-u_{z,g}\right)}$$

was used as the distance metric to define the warping path between $\hat{c}$ and $U_{v}$, where *Z* is the total number of dimensions in each signal and the total cost along the warping path for $U_{v}$ was defined as

(3) $$d_{DTW}=\sum_{\begin{array}{c} f\in ix\\ g\in iy \end{array}}d_{fg}\left(\hat{c},U_{v}\right),$$

and ix and iy represent the monotonically non-decreasing indices of $\hat{c}$ and $U_{v}$ after arrays have been respectively aligned using $d_{fg}\left(\hat{c},U_{v}\right)$ to minimize the distance between $\hat{c}$ and $U_{v}$. The DTW algorithm starts each path at $d_{11}\left(\hat{c},U_{v}\right)$, ends the path at $d_{FG}\left(\hat{c},U_{v}\right)$, and requires that paths are a combination of the following “chess king” moves—vertical: (f,g)→(f+1,g), horizontal: (f,g)→(f,g+1), and diagonal: (f,g)→(f+1,g+1). The warping path, thus, defines a mapping of monotonically non-decreasing array elements in $\hat{c}$ and $U_{v}$, where indices may be repeated any given number of times as necessary. The DTW algorithm was run in MATLAB without constraints on the number of samples the warping path could deviate from the diagonal to converge on the optimal fit between $\hat{c}$ and $U_{v}$. DTW was then applied to HR data recorded in the second session for each participant.

Statistical Analyses. Data were analyzed in MATLAB. Descriptive statistics were calculated as mean (standard deviation), median (interquartile range), and percentages (frequencies). To test the stability of heart rate responses and the percentage of time spent at a moderate-to-vigorous PA intensity during the Dance for PD^®^ class across repeated exposure to the same ≈60 min video recording, the intraclass correlation coefficient (ICC; two-way, random effects absolute agreement) was calculated between HR values observed at time points T1 (session one) and T2 (session two).

A linear mixed effects (LME) model [38] was used to test for differences between the average %HR_max_ reached at T1 and the percent of time spent engaged in moderate-to-vigorous PA during the Dance for PD^®^ class, as respectively calculated using the Rosenfeldt and Gellish equations. In the first LME model, the average %HR_max_ reached at T1 was regressed on HR equation (i.e., Rosenfeldt versus Gellish), with HR equation effect coded, adjusting for the random effect of person to account for nested observation within subjects. A second LME model was fit to evaluate the percent of time spent engaged in moderate-to-vigorous PA at T1, which was also regressed on HR equation, where the competing HR equations were effect coded, adjusting for the random effect of person.

*K*-means clustering [39] was used to automatically group heart rate responses into lower- versus higher-intensity PA segments using the array of aligned HR values from T1 as the input. The array of HR responses used as the input for the *k*-means clustering algorithm was calculated using the %HR_max_ values obtained from the Rosenfeldt equation, and wherever %HR_max_ values were ≥64% of the age-predicted HR_max_, %HR_max_ values were set equal to 1, and they were set equal to 0 otherwise for each participant. Lower- versus higher-intensity 1 s epochs were returned from the *k*-means algorithm, using a 2-group clustering solution, with the Hamming distance

(4) $$q_{Hamming}\left(a,b\right)=\frac{1}{w}\sum_{l=1}^{w}I\left(a_{l}\neq b_{l}\right),$$

where *I* is the indicator function. The *k*-means solution was returned as a vector of binary labels that represented two PA intensity clusters (i.e., lower- versus higher-intensity epochs), and the cluster solution was evaluated using a silhouette plot.

A final LME model was also used to test for differences in the mean %HR_max_ reached in the lower- versus higher-intensity Dance for PD^®^ class segments using the ML-derived labels generated from the *k*-means clustering solution. Participants’ %HR_max_, as obtained from the Rosenfeldt equation, was regressed on the binary PA intensity labels generated from the k-means solution, adjusting for the random effect of person. Given that the sample size was n<50, all LME models were fit using residual maximum likelihood [38]. LME assumptions of homogeneity of variance and normality in the residuals were evaluated using diagnostic plots, and the assumptions were found to be tenable. Model coefficients (β), and their 95% confidence intervals (95% C.I.), are presented for the LME models. The significance level was set *a priori* at α=0.05.

Qualitative movement analysis. Rich qualitative descriptions were generated for motor behaviors that were performed in the Dance for PD^®^ video recording using Telara^™^ Adaptive Learning Engine v1.0 (Developer, McCullough). Telara^™^ is a software application that utilizes a vision-language model (VLM)-based approach for the analysis of human motor behavior in image sequences. Video frames associated with respectively lower versus higher PA intensities, as determined using the PA intensity labels generated by the *k*-means solution, were separately uploaded into Telara^™^, and a comparative analysis was conducted between the motor behaviors performed in the portions of the video that were respectively labeled as lower- versus higher-intensity. A total of 20 evenly spaced frames were respectively extracted from the ≈60 min video from the lower and higher intensity PA segments, resulting in two panels of image thumbnails with 20 frames each. Prior research on qualitative motor behavior analyses conducted using a codified movement analysis framework revealed that inter-rater reliability in such applications may be low even when conducted by expert analysts [40], which suggests that no quantitative gold standard exists against which descriptions of complex motor behaviors, such as those observed in dance, can be evaluated. Therefore, experts in movement analysis (AKM and DL) directly reviewed the output from Telara^™^ against the input video frames for fidelity.

## 3. Results

As shown in Table 1, most of the participants in the sample were female and self-reported their race/ethnicity as white and not Latinx. Feasibility data showed that, of the N=19 participants who provided informed consent, n=2 participants were lost to follow-up after providing consent. HR data were unavailable for n=2 participants due to device malfunction, and n=1 participant did not complete the resting HR monitoring procedure due to technical difficulties with the device. Thus, HR data were available for n=14 participants for the Rosenfeldt HR equation, which requires a measure of resting HR, and n=15 participants had sufficient data for the Gellish HR equation, which does not require resting HR. Among the participants who provided a resting HR measure, all were able to record and save the HR data with ease at T1 and T2. Participants were able to independently put the device on and take the device off after each session; however, one participant required the help of a care partner to put on the device. Finally, the research team observed that one participant fell asleep during the online Dance for PD^®^ session, and the participant reported this was due to PD symptom-related daytime drowsiness and not a reflection of their interest.

### 3.1. Heart Rate and Physical Activity Intensity Across Sessions

When using the Rosenfeldt HR_max_ equation to compare the average %HR_max_ reached at T1 (65%; range 48% to 78%) and T2 (65%; range 51% to 75%), HR_max_ was found to be stable (ICC = 0.93). Similarly, the average percentage of time spent engaged in the class at a moderate-to-vigorous PA intensity was found to be stable (ICC = 0.77) between T1 (54%; range 0% to 100%) and T2 (62%; range 0% to 100%). The mean within-subjects difference between sessions for the Rosenfeldt equation was 0.67% (2.9) for HR_max_ and 7.1% (25.7) for the percentage of time spent at a moderate-to-vigorous PA intensity.

For the Gellish HR_max_ equation, the average %HR_max_ at T1 was 55% (range 38% to 64%) and 55% (range 41% to 64%) at T2, showing again that HR was stable across sessions (ICC = 0.95). The percentage of time spent engaged in moderate-to-vigorous PA was also stable (ICC = 0.82) when using the Gellish HR_max_ equation, with 8% (range 0% to 46%) of time spent at a moderate-to-vigorous PA intensity at T1, on average, and 10% (range 0% to 51%) at T2. The mean within-subjects difference in %HR_max_ between sessions for the Gellish equation was 0.54% (2.3), and the mean difference was 2.1% (9.5) for the percentage of time spent at a moderate-to-vigorous PA intensity.

LME results showed that the average estimated %HR_max_ was significantly lower (−10%, 95% C.I. −11.7 % to −8.7%; *t*_*df*_: −13.40_56_; *p* < 0.001) for the Gellish equation when compared to the Rosenfeldt equation (Model Adjusted *R*^2^: 0.91). The percentage of time spent at a moderate-to-vigorous PA intensity was also significantly lower (−49%, 95% C.I. −60.0% to −37.8%; *t*_*df*_: −8.84_56_; *p* < 0.001) for the Gellish equation when compared to the Rosenfeldt equation, on average (model-adjusted *R*^2^: 0.72).

#### 3.1.1. Automated Detection of Group-Level PA Intensity

**Figure 2:**
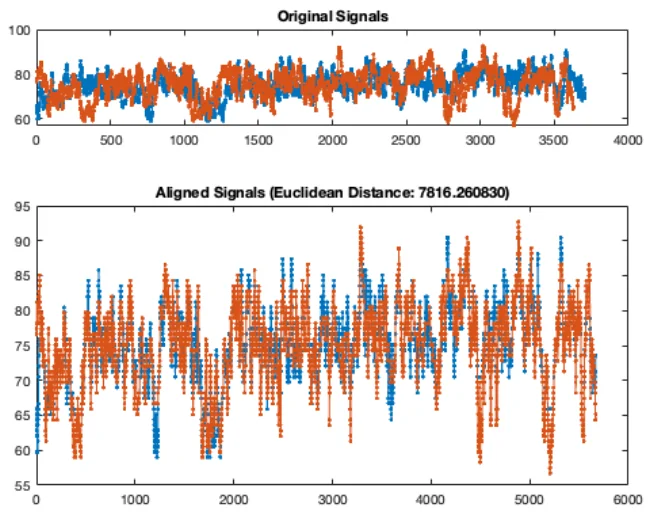
Dynamic time warping results for a single subject, with the original (**above**) and aligned (**below**) kinematic (blue) and heart rate (orange) signals between the video-derived kinematic data from the teacher and the wearable sensor-derived physiological signal from a single class participant.

**Figure 3:**
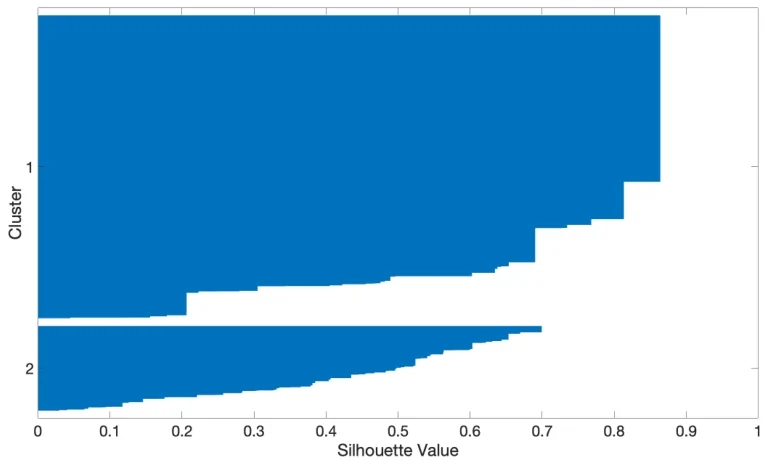
Silhouette plot for the two cluster *k*-means solution for identifying lower (class 1) versus higher (class 2) physical activity intensity segments from heart rate data collected during an online Dance for PD^®^ class.

**Figure 4:**
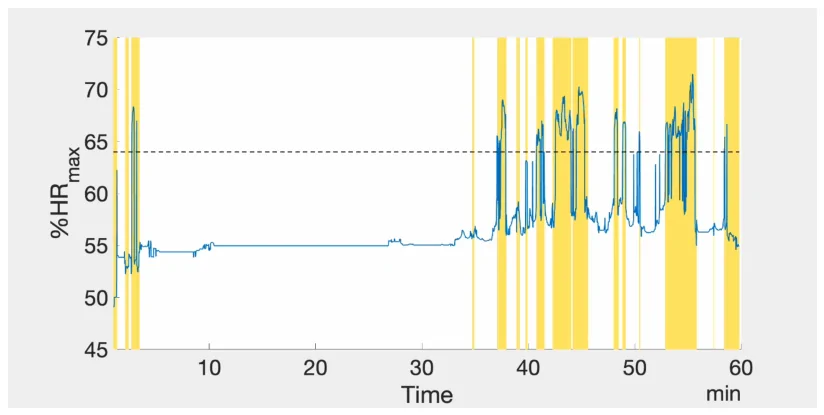
*K*-means clustering solution for identifying lower versus higher physical activity intensity Dance for PD^®^ class segments from heart rate data. The lower PA intensity class segments are shown in white, and higher PA intensity segments are shown in yellow. The average minute-by-minute heart rate response for all class participants is indicated by the blue trace, with a dotted line indicating the moderate-to-vigorous physical activity intensity cut point. Abbreviations: Minute (min), percentage of the age-predicted maximal heart rate (%HR_max_).

Figure 2 shows an example of the DTW results for a single subject with PD in relation to the kinematic data derived from the video of the Dance for PD^®^ teacher. Figure 3 displays the silhouette plot for the two cluster *k*-means solution, where cluster 1 represents the lower-intensity PA intensity segments of the class and cluster 2 represents the higher-intensity class segments. The results for the *k*-means clustering solution are shown in Figure 4.

#### 3.1.2. Comparison of *k*-means Derived PA Intensity Segments

Table 2 shows differences between the lower- and higher-intensity class segments based upon the PA intensity labels obtained from the *k*-means solution. The mean %HR_max_ during the higher-intensity lesson plan activities was 5.5% greater, on average, than during the lower-intensity lesson activities (model-adjusted *R*^2^: 0.82).

### 3.2. Automated Qualitative Motor Behavior Analysis

**Figure 5:**
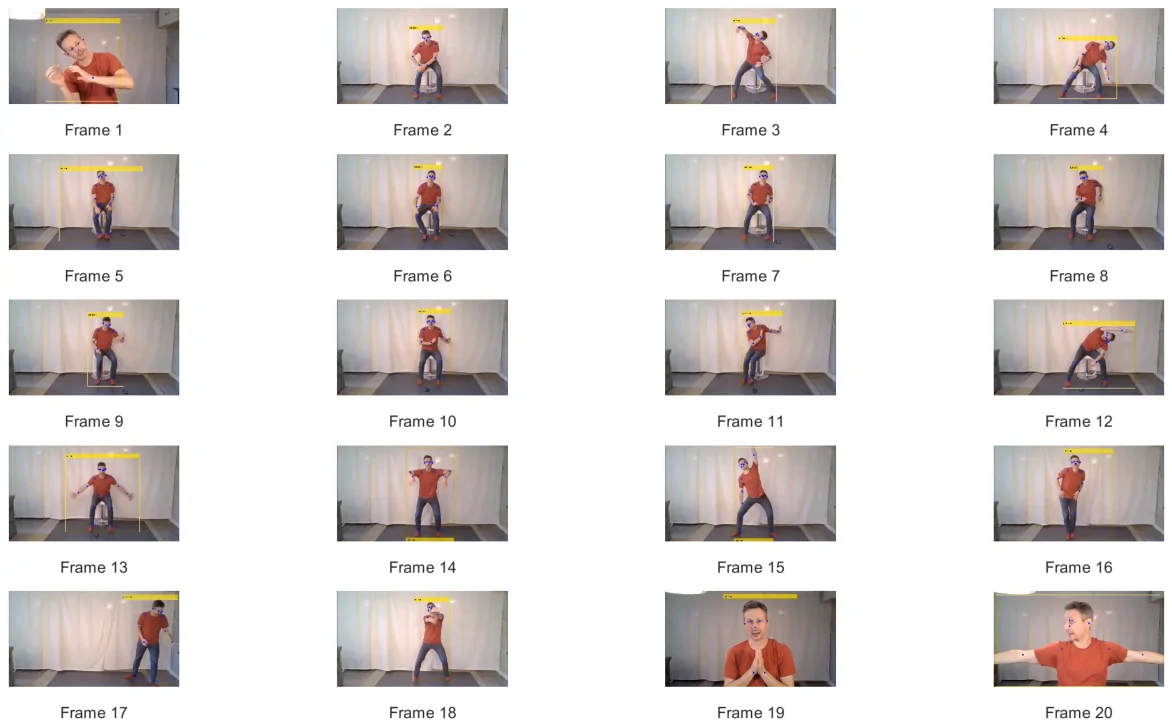
Extracted key frames from the lower-intensity physical activity Dance for PD^®^ class segments (Panel 1) defined by the *k*-means clustering solution, as provided to Telara^™^ Adaptive Learning Engine for automated qualitative motor behavior analyses. Bounding boxes (yellow) and keypoints (blue) are shown.

**Figure 6:**
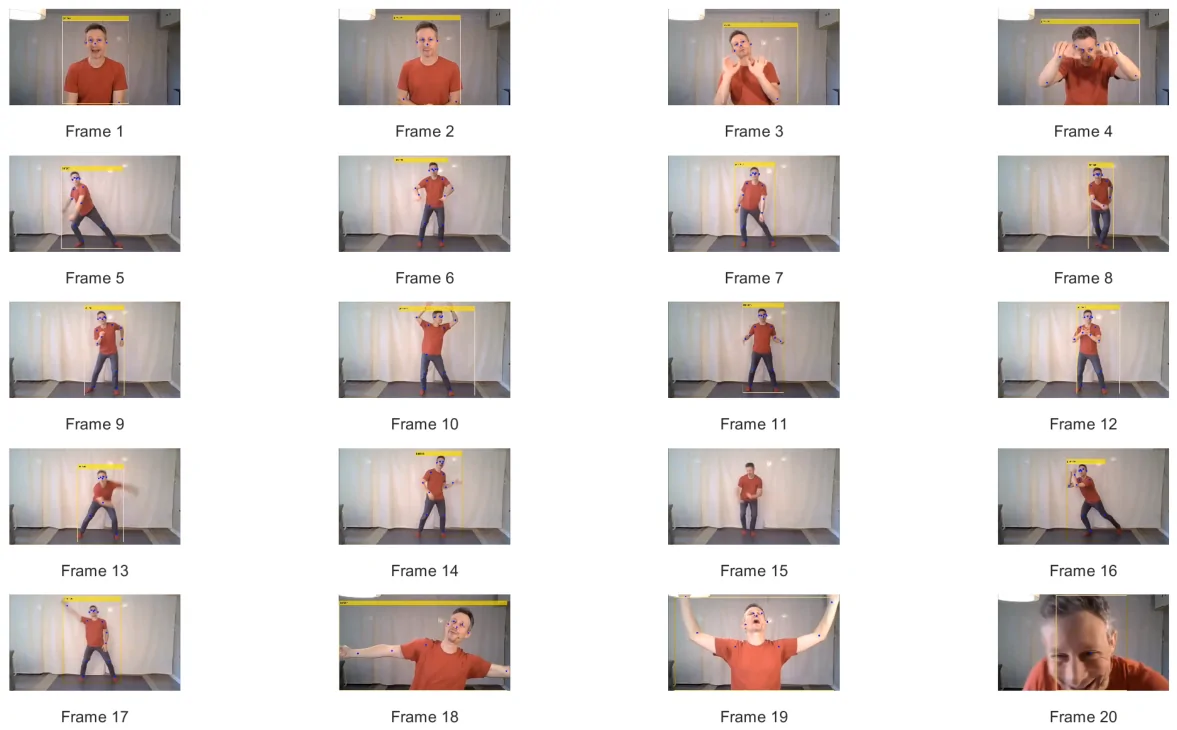
Extracted key frames from the higher-intensity physical activity Dance for PD^®^ class segments (Panel 2) defined by the *k*-means clustering solution, as provided to Telara^™^ Adaptive Learning Engine for automated qualitative motor behavior analyses. Bounding boxes (yellow) and keypoints (blue) are shown.

The results from Telara^™^ showed that the motor behaviors observed in the lower- (Figure 5) versus higher-intensity (Figure 6) lesson plan segments, as defined by the *k*-means clustering solution, differed qualitatively.

#### 3.2.1. Viewing Conditions

Telara^™^ reported that the same individual was observed wearing a short-sleeved rust/orange shirt, dark jeans, and red-orange sneakers across the two respective 20-frame image panels. The subject in the video was observed from an anterior view, with a few frames offering an oblique view of the subject. The subject was observed to be upright (standing), with several seated frames. The subject was reported to be in an indoor studio setting, with a plain light-colored fabric backdrop and a gray and white striped floor or mat on the floor. The report noted that the size of the images in the panels, which were comprised of 20 small thumbnail frames, rendered some specific details more difficult for Telara^™^ to confirm.

#### 3.2.2. Motor Behavior Comparison

The report from Telara^™^ showed that non-weight-bearing arm movements were the dominant action observed in both panels, and that both opening the limbs away from the midline and trunk rotation were also observed across panels. Within Panel 1 (lower intensity PA), Telara^™^ observed that the subject transferred weight between being seated on the stool and standing; the subject brought their hands together toward the body’s midline; the subject also appeared to engage in some static or held positions. Within Panel 2 (higher intensity PA), Telara^™^ observed transfer of weight from a narrow to wide base of support during lunges; the subject engaged in actions that required balance; and the subject turned the head/neck from side to side.

Telara^™^ conducted a body segment-specific comparison of the motor behaviors observed within Panel 1 and Panel 2, as shown in Table 3. The report highlighted key differences and similarities in movement quality and patterns between Panel 1 and Panel 2. In particular, Telara^™^ observed within Panel 2 more dynamic actions, and a wider base of support as compared to Panel 1 which featured a narrower base of support and more upright standing/seated positions. Panel 2 showed more visible trunk rotation and forward lean, while the trunk appeared to stay closer to upright more often in Panel 1. In Panel 1, there were seated activities that took place on a stool that were not present in Panel 2, and Panel 2 included some tight close-up frames of the face that do not appear in Panel 1. With respect to similarities between Panel 1 and Panel 2, Telara^™^ observed that both panels strongly featured expressive arm gestures rather than walking, running, or jumping; both panels included moments with the arms open and spreading away from the midline of the body, and both panels included trunk rotation while the arms reach across the body or to the side.

A direct excerpt from the Telara^™^ comparative motor behavior analysis summary report is as follows: “Panel 1 and Panel 2 capture the same performer producing two different `flavors’ of expressive, gesture-driven movement in the same studio setting—Panel 1 built around a narrower, more upright standing/seated base with communicative hand gestures, and Panel 2 built around a wider, lunging standing base with more trunk rotation, forward lean, and close-up facial framing.”

#### 3.2.3. Telara^™^ Fidelity

Comparison of the output from Telara^™^ with motor behavior analyses conducted by human experts showed that Telara^™^ maintained high fidelity in its comparative analysis of the 40 thumbnail frames. Only minor errors in the output from Telara^™^ were detected, namely that the subject appeared to be wearing sweatpants and socks when observed in the video, as opposed to dark jeans and shoes as reported by Telara^™^. In Panel 1 (Frame 17), the subject was reaching off-camera to control the music, but the audio controller was out of frame. However, Telara^™^ appropriately noted that the size of the thumbnails somewhat limited its ability to determine some of the finer details, such as the subject’s clothing, in the image sequences. As can be seen in Figure 5 and Figure 6, the output from Telara^™^ otherwise maintained high fidelity, relative to the input images, was comprehensive in its scope, and appropriately identified major motor behavior pattern differences between the respective panels without receiving any context about the motor behavior intensities represented in each panel of images nor any information about the underlying goals of the image analysis.

## 4. Discussion

In this feasibility study, we found that middle-aged to older adults with PD engaged in a ≈60 min online Dance for PD^®^ class at a moderate PA intensity on average; however, wide variability in participant responses to the online class was observed. At the group level, 58% of the class was spent at a moderate PA intensity, though some adults did not engage in any moderate-to-vigorous PA during the online class. Despite individual variability in HR responses to the online dance class, we found that both the mean PA intensity and the percentage of time spent engaged in the class at a moderate PA intensity were stable within participants when repeatedly exposed to the same video stimulus. At the same time, our feasibility study identified challenges in the use of technology among some middle-aged to older adult participants, thus leading to important findings that may inform future study designs that intend to use wearable devices for quantifying HR remotely in this population group. Importantly, we found that a signal processing pipeline that utilized both unsupervised machine learning and a vision-language model was able to automatically align asynchronous HR data across participants, identify class segments that respectively reflected lower- versus higher-intensity PA at a group level using HR data, and provide rich qualitative descriptions about the differences and similarities between the motor behaviors demonstrated in the lower- versus higher-intensity lesson plan activities within a ≈60 min video. Taken together, our feasibility study demonstrated that, despite some challenges with initial signal acquisition using wearable devices among a population group of middle-aged to older adults, a multimodal data-driven pipeline can robustly identify which motor behaviors within a complex mode of PA, such as dance, were associated with class participants reaching a health-enhancing PA intensity at a group-level.

A review of field notes collected throughout this study revealed several important considerations for future studies that wish to collect HR data remotely from middle-aged to older adults by deploying wearable sensor kits. Some participants in this study reported using an iPad for the first time when they received the wearable sensor kit, and guiding some participants through the process of learning how to use the HR monitor, utilize a videoconferencing platform, use the iOS based application, and connect the iPad to WiFi so that data could be stored in the cloud was time-consuming for some participants and the research team. However, after learning how to use the wearable sensor, videoconferencing platform, and iPad, most participants were able to initialize the HR recording sessions using the iPad and wearable sensor with little intervention from the research team. One participant was unable to connect the HR monitor to the iPad, even after the research team attempted to help troubleshoot the connection issues. Another problem that emerged in the course of data collection was that updates to the HR recording application occurred during this study, which led to some connectivity issues for some participants. Finally, at least one participant noted that tremor in the upper extremity made it difficult to tighten the HR chest strap independently. Other participants noted that the chest-worn strap needed to be loosened, and these participants had to work with the research team via video conference to loosen the strap to their comfort without loosening the monitor too much, which would negatively affect HR recordings. Prior studies that have used decentralized approaches for collecting physiological data *in situ* using wearable sensors have also found that, while the approach is successful for most participants, there are those who experience barriers while using wearable technologies [41,42]. Findings from our study further confirm prior findings that middle-aged to older adults may require additional time to become familiarized with wearable technologies and videoconferencing platforms [42], but that given adequate time to become oriented to using these technologies, high-quality data can be obtained via decentralized measurement approaches in this population group. While chest-worn HR monitors generally provide more accurate data than wrist-worn sensors [43,44], additional research may be needed on the trade-offs between chest-worn and wrist-worn device placements within decentralized data collection paradigms, especially among middle-aged and older adults who may have less experience using various technologies or who may initially experience barriers when using new technologies [45].

We found that HR responses to an online Dance for PD^®^ class were stable for middle-aged to older adults with PD when repeatedly exposed to the same stimulus on different days. It was also found that the use of the Rosenfeldt equation for determining HR_max_ led to notably higher percentages of time spent at a moderate-to-vigorous PA intensity during the online dance class (54% to 62%) when compared to the Gellish Equation (8% to 10%). When using the Rosenfeldt equation, which was specifically modeled using HR data from adults living with PD, we found that the average HR response while participating in a ≈60 min online Dance for PD^®^ class placed participants at a relative PA intensity ≈65% of their HR_max_, on average, which meant participants were engaged in the online dance class at a moderate PA intensity at the group level. We also found that, at the group level, ≈34 min of the online class was spent at a moderate-to-vigorous PA intensity, which represents roughly 22% of the minimum weekly health-enhancing PA volume recommended for adults [25,46]. It was also observed, however, that some participants did not reach moderate PA intensity during the online dance class. For example, a participant informed the research team that the songs used during the class were relaxing, and the participant reported falling asleep during the class due to PD symptom-related daytime drowsiness, rather than due to a lack of interest. To ensure that this study remained ecologically valid, the HR data from the participant who fell asleep were not excluded. A previous study on HR during a 90 min in-person dance class for adults with PD reported that during seated and standing activities, HR was significantly higher when students were dancing than when the instructor was teaching, though the mean difference in HR was <2 bpm [47]. Research on HR responses to PA among adults with PD has reported that, when compared to those without PD, differences in acute increases in HR in response to PA exposure were lower among adults with PD, and HR recovery responses were slower. However, after adjusting for clinical and autonomic factors, researchers found that the lower mean HR observed during PA was not independently explained by PD diagnosis, while blunted responses in HR recovery were independently associated with having a diagnosis of PD [48]. Given prior findings showing that various modes of PA differentially impact specific PD symptoms [49], and the important relationship between clinical and autonomic factors among adults with PD and HR responses to PA [48], additional longitudinal research is needed on the cumulative effects of online Dance for PD^®^ classes while expressly measuring the PA intensity, duration, and frequency of these online dance exposures and accounting for clinical and autonomic factors that may explain differences in HR responses during PA.

The novel, multimodal data analysis pipeline in this study utilized signal processing to align asynchronous HR signals collected among fourteen adults over several months with kinematic data extracted from a single instructor in an online Dance for PD^®^ class, which was followed by unsupervised machine learning for labeling group-level PA intensity into lower- versus higher-intensity PA segments using the aligned HR data and, lastly, a VLM approach for qualitatively comparing the lower- versus higher-intensity PA behaviors observed in video frames. Statistical analysis of the unsupervised learning approach for annotating image frames showed that the aligned HR data for the higher-PA-intensity class segments was significantly greater, on average, than the lower-intensity PA class segments. When using Telara^™^ to apply a VLM to extracted video frames from the respectively lower and higher PA intensity segments, as labeled using unsupervised machine learning, it was found that the motor behaviors in the lower-intensity segments featured a narrower base of support, sitting, and arm gestures, while the higher-intensity segments featured a wider base of support, standing, lunging, and greater rotation of the trunk in addition to arm gestures. A prior study using a multimodal signal processing pipeline for analyzing data obtained using force plates and markerless motion capture to identify different movement strategies during jumping reported successful identification of three injury risk profiles using a *k*-means clustering approach [50]. Other researchers have used VLMs for differentiating between activity intensities observed in video; however, researchers found that the VLMs utilized for PA classification were only able to sufficiently differentiate sedentary behaviors from PA behaviors rather than differentiate between PA intensities, and researchers reported that the VLM approach generalized poorly when applied across datasets [51]. The pipeline implemented in our study utilized unsupervised machine learning to first classify lower- versus higher-intensity PA, then it used the labels generated from the unsupervised approach to segment video frames. In tandem with evaluating the results of the unsupervised machine learning approach for identifying quantitative difference in the HR responses between the two PA intensity classes that were returned, we implemented a VLM-based approach to provide rich, qualitative descriptions of the behaviors observed across the two PA intensity classes. Thus, our study extends prior methods by providing a multimodal approach that yields both quantitative and qualitative output that comprehensively characterizes motor behavior and its physiological correlates using heart rate data and video data.

Our study delimited the scope of its investigation to a single online Dance for PD^®^ class in order to determine the stability of HR responses among adults with PD who engaged in a dance class remotely. Given the complex nature of dance behavior, which may be performed across a range of styles and contexts [10], it is therefore possible that other Dance for PD^®^ lesson plans may elicit relatively lower or higher PA intensities among participants. The population of adults who enrolled in this study was largely female, which may reflect the fact that participation in dance in the United States has historically been higher among women. Additionally, we delimited the study sample to include adults with PD without significant balance impairments for safety purposes. Therefore, a wider range of online Dance for PD^®^ classes, taught among a larger sample of adults with PD, should be analyzed using the automated multimodal data analysis pipeline applied in this study to characterize a wider number of Dance for PD^®^ classes that may utilize different styles of dance to engage participants in PA. Additionally, Telara^™^ was only provided 40 frames to respectively describe high-level differences in motor behavior between lower- versus higher-intensity class segments, and providing a greater number of frames to the VLM may have provided Telara^™^ with a wider range of motor behaviors to review and use in its comparative analysis. Nevertheless, the output from Telara^™^ maintained high fidelity with respect to the image thumbnails representing the lower- versus higher-intensity PA that were input into the software for analysis. A major strength of this study is that, to our knowledge, it is the first to investigate HR responses to an online Dance for PD^®^ class *in situ*. Thus, our study represents an important extension to prior research by providing empirical evidence on the stability of HR responses to a single class, in addition to providing evidence that an online Dance for PD^®^ class may elicit a sufficient stimulus for middle-aged to older adults to reach a health-enhancing PA intensity in remote settings. Additionally, this study appears to be the first to use a multiphased approach for automatically classifying PA intensity in response to home-based PA programming by combining physiological data collected asynchronously and kinematic data to segment video while also complementing an unsupervised machine learning approach with a VLM-based approach to describe differences in motor behavior performed during lower- and higher-intensity lesson activities.

Future research on home-based PA programming may apply the multimodal data processing pipeline featured in this study to physiological data that are collected asynchronously in order to identify physiological responses to lesson plan activities at the group level that occur in response to prerecorded classes administered by video, in addition to providing rich descriptions of the specific lesson activities associated with the observed physiological responses. Specifically, where there is already an available corpus of PA training videos, researchers and clinicians may consider asking class participants to simply share their HR data after completing a class, which can then be aligned across multiple participants using the proposed pipeline in order to determine group-level physiological responses to specific lesson activities and thereby derive the group-level PA intensity of lesson activities in tandem with qualitative descriptions of which activities may support reaching health-enhancing PA intensities while engaging in PA remotely. With respect to the study of dance behavior, and especially with regard to online Dance for PD^®^ classes, the use of the multimodal data analysis pipeline described in this study could serve to rapidly advance what is known about the PA intensity of dance behavior among adults with PD by affording researchers with an automated method for determining group-level physiological responses to online dance classes, along with descriptions of the motor behaviors differentially associated with various PA intensities, while adults engage in classes remotely.

## 5. Conclusions

In conclusion, participation in dance has been associated with improved health outcomes among adults with PD, though little is known about the PA intensities or motor behaviors that have led to the observed health benefits associated with engaging in dance. This study implemented a multimodal data analysis pipeline to better understand physiological responses during an online dance class, and the motor behavior patterns differentially associated with lower- versus higher-intensity lesson activity, among adults with PD while engaged in a ≈60 min online Dance for PD^®^ class. It was found that HR responses to an online Dance for PD^®^ class were stable across repeated exposure to the same online dance class among adults with PD. At the group level, the total time spent at a moderate-to-vigorous PA intensity represented ~22% of the minimum weekly PA dose recommended for adults with PD. The multimodal data analysis pipeline effectively differentiated lower- versus higher-intensity PA activities at a group level, in addition to providing rich descriptions of the specific motor behaviors associated with each PA intensity. Future studies may implement the multimodal approach utilized in this study to more rapidly advance what is known about the PA intensity of home-based PA programming for adults with PD, especially within the context of studying dance behavior in relation to health and well-being.

## Figures and Tables

**Table 1:** Sociodemographic information for adults (n=15) with a diagnosis of Parkinson’s disease.

	Mean (SD)
**Age** (years)	69 (10.5)
	**Median (IQR)**
**Years since PD diagnosis**	7 (5)
**Resting HR** (bpm; *n* = 14)	77 (25)
**Daily activities** (*n*)	8 (3)
	**% (** ***n*** **)**
**Female**	93.3% (14)
**Race**	
White	93.3% (14)
Black	6.7% (1)
**Ethnicity**	
Not Latinx	93.3% (14)
Latinx	6.7% (1)

Abbreviations: Parkinson’s disease (PD), beats per minute (bpm).

**Table 2:** Differences in heart rate across lower- versus higher-intensity lesson activities during an online Dance for PD^®^ class among adults (n=14) with PD.

	β [95% C.I.]	t_*df*_	*p*-Value
Lower-Intensity Activities (%HR_max_)	56.97 [53.71 to 60.23]	34.29_50258_	<0.001
Higher-Intensity Activities (%HR_max_)	5.52 [5.46 to 5.59]	168.05_50258_	<0.001

Note: Lower- versus higher-intensity lesson activities were identified using an unsupervised machine learning approach. Abbreviations: Parkinson’s Disease (PD), percentage of age-predicted maximal heart rate (%HR_max_).

**Table 3:** A qualitative comparison of motor behaviors between lower- and higher-intensity lesson plan activities by body segment, as reported by Telara^™^ Adaptive Learning Engine.

Body Region	Panel 1	Panel 2
Feet/Ankles	Neutral or wide stance; also seated with feet planted	Frequently staggered (lunge-type) stance
Knees/Legs	Straight to soft-bent standing; ~90° bent when seated	Unevenly bent (front knee more, back knee less)—lunge pattern
Pelvis/Hips	Mostly square to the camera	More rotation relative to the shoulders in several frames
Spine/Trunk	Some forward/side leaning	More forward leaning and rotation overall
Shoulders/Arms	Wide range: out to the side, overhead, back to the body	Wide range: overhead, out to the side, across the body
Elbows	Mix of bent/straight	Mix of bent/straight
Wrists/Hands	Open (spread) and closed (pressed together) shapes	Mostly open/reaching shapes; some near the face
Head/Neck	Mostly facing forward	More turning and tilting, especially in close-ups

Table 3 provides a direct excerpt of an vision-language model-generated comparative motor behavior analysis, as reported from Telara^™^ Adaptive Learning Engine.

## Data Availability

The aggregate data presented in this study are available on request from the corresponding author.

## Abbreviations

The following abbreviations are used in this manuscript:

PA	Physical activity
PD	Parkinson’s disease
HR	Heart rate
VLM	Vision–language model
